# Binary PSO with Classification Trees Algorithm for Enhancing Power Efficiency in 5G Networks

**DOI:** 10.3390/s22218570

**Published:** 2022-11-07

**Authors:** Mayada Osama, Salwa El Ramly, Bassant Abdelhamid

**Affiliations:** 1 Electronics and Communications Department, Faculty of Engineering Science and Arts, Misr International University, Cairo 11828, Egypt; 2 Electronics and Communications Department, Faculty of Engineering, Ain Shams University, Cairo 11517, Egypt

**Keywords:** 5G HetNets, BPSO, classification trees (CTs), soft frequency reuse (SFR), small cells (SCs)

## Abstract

The dense deployment of small cells (SCs) in the 5G heterogeneous networks (HetNets) fulfills the demand for vast connectivity and larger data rates. Unfortunately, the power efficiency (PE) of the network is reduced because of the elevated power consumption of the densely deployed SCs and the interference that arise between them. An approach to ameliorate the PE is proposed by switching off the redundant SCs using machine learning (ML) techniques while sustaining the quality of service (QoS) for each user. In this paper, a linearly increasing inertia weight–binary particle swarm optimization (IW-BPSO) algorithm for SC on/off switching is proposed to minimize the power consumption of the network. Moreover, a soft frequency reuse (SFR) algorithm is proposed using classification trees (CTs) to alleviate the interference and elevate the system throughput. The results show that the proposed algorithms outperform the other conventional algorithms, as they reduce the power consumption of the network and the interference among the SCs, ameliorating the total throughput and the PE of the system.

## 1. Introduction

**Figure 1:**
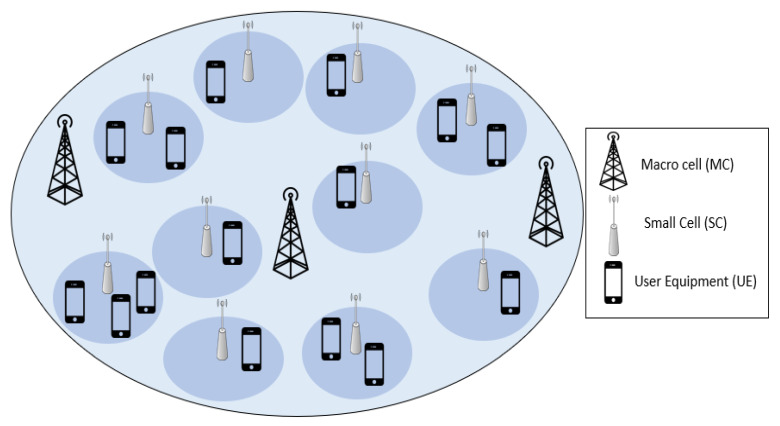
General representation of HetNet scenario with densely deployed small cells.

Recently, the exponential growth of the numerous wireless devices and the data-hungry applications have earned huge significance. This required imperious expansion of the 5G network to support the forthcoming 5G use cases, such as video live-streaming, conferencing, online gaming, etc. [1,2]. Moreover, the 5G cellular network is planned to elevate the capacity 1000 times and the spectrum efficiency by 5–15 times with respect to 4G [3,4]. This can be achieved by utilizing heterogeneous networks (HetNets), which can enhance the system data rates and the quality of service (QoS) of the users as the small cells (SCs) are deployed within the macro cells (MCs) coverage area. Furthermore, SCs offer the benefit of providing service to previously uncovered regions and in the network regions demanding larger capacity [2,3,5,6,7]. Figure 1 shows a general representation of the HetNet scenario with the MCs underlaid by the densely deployed SCs.

It is foreseen that the massive growth in the SC deployment will be continued in the coming years [8,9], leading to various challenges such as the interference among the SCs [10], their elevated power consumption [11,12], and the elevated operating expenses [9]. Thus, it is crucial to face these challenges to ameliorate the performance of 5G HetNets.

The main objective of our paper is to propose a new approach for the irregular nature of the 5G HetNets that merge the usage of both binary particle swarm optimization (BPSO) algorithm with linear increasing inertia weight (IW) and soft frequency reuse (SFR) to maximize the power efficiency (PE) of the SCs and minimize the number of active SCs while guaranteeing the QoS for the UEs. In SFR, every SC is split into center and edge regions where one of the unutilized sub-bands by the edge regions of the adjoining SCs is allocated to the edge region of the SC, while the remaining sub-bands are utilized in the center region of the SC with reduced transmission power to diminish the interference to the adjoining SCs. Unlike the prior works [9,13,14] that will be discussed later in Section 2, the proposed algorithm utilizes the linear increasing IW-BPSO algorithm for selecting the SCs to be switched on/off, and we propose the SFR utilizes classification trees (CTs) to enhance the network PE, taking into consideration the irregular nature of the 5G HetNets. The on/off switching of SCs using BPSO algorithm is carried out first; then the SFR is applied for sub-band allocation to minimize the number of operations required for allocating the sub-bands to the SCs.

The main contributions of this paper can be summarized as follow:

1. Propose an algorithm for irregular 5G HetNets based on BPSO algorithm for SC on/off switching to ameliorate PE of the system, and, using a linearly increasing IW approach where the IW is linearly increasing in each iteration, to enhance the convergence of the BPSO algorithm.
2. Propose a novel frequency allocation algorithm for SFR based on the CTs as it is simple and accurate machine learning (ML) technique to mitigate the interference among the irregularly shaped SCs.

Results demonstrate that the proposed algorithms have superior performance over the conventional algorithms (always on, random 10%, and BPSO only), as it has higher total system throughput and PE, and lower system power consumption and outage probability.

The remainder of the paper is organized as follows: the literature review is presented in Section 2. Section 3 demonstrates the system model, and Section 4 explains the proposed algorithms. Then, Section 5 shows the simulation results. Eventually, Section 6 concludes the paper.

## 2. Literature Review

Recently, immense research has been carried out to ameliorate the PE of the mod-ern communication networks, such as satellite and terrestrial networks [15,16], massive MIMO systems [17] and SCs networks [18,19,20,21,22,23].

SC on/off switching is an auspicious approach to minimize the power consumption and enhance the PE of the system [18,19,20,21,22,23]. The authors in [18,19] studied the elevated the energy consumption of WLAN. They proposed solving the problem by on/off switching and power adjustment of the access stations. The authors in [20] proposed a load-aware strategy, where SCs in HetNets are switched to sleep mode according to their load level. In [21], every SC independently switches off upon the decrease in the number of user equipments (UEs) and activates using one of three approaches. The first approach is the sleeping SC keeps sensing the interference plus noise levels and switches on when a new UE is sensed in its coverage area. The second approach is that the MC sends a wake-up request to all SCs upon increasing the number of UEs associated with the MC, then switches off the SCs with no UEs later. In the third approach, a time advance indicator is sent to the MC by the UEs and the SCs and is utilized by the MC to determine the nearest SC to the UE to switch on. In [22], pre-sleeping SCs at the same zone create a sleeping cluster. Then, the SCs in the sleeping cluster are randomly selected to be switched off leaving only one active SC to guarantee the coverage. On the other hand, the authors in [23] proposed switching off the SCs and handing over the UEs to the MC. However, the unplanned SC off switching may increase the unnecessary handovers and underutilize the SCs. Thus, novel SC on/off switching techniques are required to enhance the performance of the 5G HetNets and to reduce the power consumption of the system.

PSO is a prevalent meta-heuristic algorithm used in solving optimization problems [9]; thus, the authors in [9,24,25] utilized the PSO algorithm for SC switching to enhance the performance of the system. The authors in [9] proposed an efficient cell modeling (ECM) algorithm to set up the connection initially between the UEs and the SCs by selecting the strongest received signals. Then, the BPSO algorithm is utilized to turn off the excessive SCs. The authors in [24] proposed first utilization of BPSO algorithm to choose the MCs’ optimum locations not only to achieve minimum overlap but also to guarantee a reasonable coverage for the UEs. Then, a multi-stage PSO (MS-PSO) algorithm consisting of two interactive loops are utilized. The outer loop is utilized to switch the SCs (on or off), while the inner loop is utilized to optimize the active power of the SC and to elevate the power of the SC if the data rate rises. On the other hand, a combined optimal frequency and power allocation (COFPA) scheme is proposed in [25]. First, using the BPSO algorithm, the MCs are switched on and off until the lead interferer is abolished with minimum cost function and with a reasonable coverage to the UEs. Then, the MS-PSO algorithm is utilized to control the SC switching to mitigate the interference and minimize the power consumption. However, the convergence of the PSO can be enhanced by adjusting its IW, leading to improve the system performance. 

IW has a significant role in the process of offering a trade-off among diversification and intensification skills of PSO algorithm. Reducing the IW facilitates exploring the search space (global search), although raising the IW aids exploiting the search space (local search) [26] to find the solution (particle). Numerous approaches are presented to adjust the IW such as the constant IW [9,27] and the random IW [28]. However, the constant IW approach can fail to balance exploration and exploitation because of the lack of adjustment of IW [29,30]. On the other hand, the authors in [31] propose a linearly decreasing IW technique where the IW is initialized at a larger value; then it is linearly reduced to a smaller value. However, in this technique, the tendency of the particles to local search is constantly increasing. The authors in [32,33] demonstrated that the increase in IW surpasses the decrease in IW for PSO on all their tested benchmarks. Thus, the linearly increasing IW is adopted in our paper. 

Various interference mitigation techniques for the modern networks are presented in the literature such as advanced multiple access techniques [34,35] and frequency reuse (FR) [13,36,37,38,39]. FR is an auspicious approach aiming to mitigate the interference in the modern HetNets [37]. SFR is presented to minimize the interference in HetNets [13,36,38,39]. The authors in [36] present SFR in HetNets, where the MC organizes the assignment of the resource plans to the SCs, and the SCs choose the resource plan. However, this algorithm cannot be implemented in the absence of the MC. A multi-level SFR (MSFR) for HetNets is demonstrated in [38], where every cell is split into three zones (central, intermediate, and edge), utilizing various frequency segments and transmission power levels. The authors in [13] proposed a novel SFR algorithm to mitigate the interference by splitting the SC to the center and edge regions. Moreover, the on/off switching of the SCs depends on their interference contribution rate (ICR) values. The authors in [39] presented an MSFR scheme where every MC is split into various circular areas, and different spectrum and power are allocated to every area. Unfortunately, in this scheme, SFR is not applied to the SCs but only the MCs. New SFR approaches are essential to alleviate the interference and improve the network throughput in 5G HetNets.

Moreover, the performance of 5G HetNets can be enhanced using ML techniques [40]. Decision trees (DTs) is one of the propitious supervised learning (SL) techniques, where every training example must be fed with its label to train a learning model, then utilize this model to predict the output for any new data. The authors in [41] proposed using supervised ML to enhance both the classification of services and the distribution of network resources in 5G networks. Moreover, the results reveal that DTs and random forests are the best approaches. DTs are split to classification trees (CTs) and regression trees [40]. The authors in [42] studied several anomaly detection techniques in 5G traffic. The performance of these techniques is analyzed based on multiple factors such as the probability of identifying anomalies and the probability of detecting a false positive. The results demonstrated that the CTs technique outperforms the other techniques. The problem of monitoring and predicting the quality of experience of cellular networks is studied in [43]. The authors compared various SL techniques and trained them utilizing training data based on traffic measurements of the UEs from a field trial study. The CTs are the ultimately chosen model because of their superb prediction accuracy and their prediction speed. In our paper, CT is utilized for the first time to allocate the frequencies in the SFR 5G network.

Finally, to match real-life scenarios, the irregular nature of the 5G HetNets was modeled utilizing Voronoi cells [44,45,46,47]. Because of the immense deployment of SCs, Voronoi cells are considered more practical than traditional hexagonal grids [48].

## 3. System Model

Consider 5G HetNet with densely deployed Voronoi SCs, where the SCs are deployed within the MCs coverage area. In this scenario, the MCs and the SCs utilize different frequency bands, mitigating the cross-tier interference between them. The MCs stay active to maintain the coverage of the network when the SCs are turned off. On the other hand, the SCs are either active (on) or asleep (off). When the SC is turned off, regular discovery signals are sent by the SC to be detected by any potential user. Each UE reports its channel state information and its reference signal received power (RSRP) to its SC. The SC sub-band allocation and the SC switching is organized by a main controller, or the MC if the main controller is absent, to collect the data from the SCs, allocate the sub-bands to the SCs, and determine the on/off switching decisions of the SCs. Furthermore, it is assumed that all UEs in every SC are located inside the coverage area of the SC. In case of the existence of any coverage holes in the SCs, relay nodes can be utilized to cover these holes [49]. However, this is not considered in our paper. Nomenclature lists the described symbols utilized in this paper.

An SC on/off indicator γ  is defined, where $\gamma_{m}=1\;$ when the SC *m* is active; otherwise, $\gamma_{m}=0$. The number of SCs is denoted by “*M*” and the number of UEs in SC *m* is denoted by “$U_{m}$”. The UE association indicator $\varphi_{m,k}=1$ if UE *k* is associated with SC *m*; otherwise, $\varphi_{m,k}=0$. The signal to interference and noise ratio (SINR) of UE *k* in SC *m* can be calculated as [14]:

(1) $$SINR_{k,m}=\frac{\gamma_{m}P_{m}G_{m,k}}{\sum_{n\;\neq m,\;\;n\;\epsilon \;M}\gamma_{n}P_{n}G_{n,k}+N}$$

where $P_{m}$ and $P_{n}$ are the transmission powers of the serving SC *m* and the interfering SC *n*, respectively. The channel gain between UE *k* and serving SC *m* is $G_{m,k}=d_{m,k}{}^{-\alpha }$, where $d_{m,k}\;$ is the distance between SC *m* and UE *k* and α is the path loss exponent [50]; the channel gain between UE *k* and interfering SC *n* is $G_{n,k}$ and *N* is the noise power. The data rate $R_{k,m}$ of UE *k* in SC *m* is also calculated by Shannon’s formula as [13]:

(2) $$R_{k,m}=B_{k}\;\log_{2}(1+SINR_{k,m})$$

where $B_{k}=B_{RB}L_{k,m}$ is the bandwidth allocated to UE *k*, while the resource block (RB) bandwidth is $B_{RB}$ and $L_{k,m}$ is the number of requisite RBs for UE *k* in SC *m* to achieve the minimum data rate [14].

While the total throughput of the system is given by [9]:

(3) $$C_{sys}=\sum_{m=1}^{M}\gamma_{m}\sum_{k=1}^{U_{m}}R_{k,m}$$

Due to the dense deployment of the SCs, some SCs can be turned off without affecting the QoS of the UEs. Thus, the total power consumption of SC *m* can be calculated as [13]:

(4) $$P_{m_{tot}}=\beta P_{m_{on}}+\left(1-\beta \right)P_{m_{on}}\gamma_{m}+\theta_{m}P_{m_{tx}}\gamma_{m}$$

where $P_{m_{on}}$ and $P_{m_{tx}}\;$ are the baseline and the transmission power consumption, respectively, while β  is the inactive level of the SC, such that $P_{m_{off}}=\beta P_{m_{on}}$, and $\theta_{m}\;$ is the portion of power consumption that is due to the feeder losses and power amplifier of SC *m* [51]. The total power consumption of the system ($P_{sys}$) is the sum of the power consumption of all SCs. The PE of the system is given by [9]:

(5) $$PE_{sys}=\frac{C_{sys}}{P_{sys}}$$

To improve the PE, the SCs on/off switching decisions using BPSO is proposed in this paper. The PSO is an iterative population-based search algorithm inspired by the hunting behavior of a flock of flying birds [52,53,54]. In PSO, every particle is considered a bird of the flock and represents a possible solution to the problem [30,55,56,57]. The search begins with an initial set of particles and attempts to find the best solution by searching around the solution space. The motion of the particle is based on its local best position, and the best-known position of all the other particles [9,52]. The fitness value of every particle is calculated using a fitness function that is optimized in every iteration [9,52]. 

The set of particles X = {$x_{1},\;x_{2},\;\ldots ,\;x_{N_{par}}$} is defined, where $x_{N_{par}}$ represents one possible status for the SCs, while $N_{par}$ is the swarm size. In BPSO algorithm, the population is randomly initialized as binary values. For every particle, the population binary value of 1 signifies the active SC, while 0 signifies the sleeping SC. The velocity of the particle *j* is initialized as [9,58]:

(6) $$v_{j}=v_{min}+\left(v_{max}-v_{min}\right)\;a_{1}$$

where $v_{min}$ and $v_{max}\;$ denote the minimum and the maximum velocity of the particle, respectively, while $a_{1}$ is a random number uniformly distributed between 0 and 1 [9]. The velocity and the position of the particles are updated in each iteration. The velocity of the particle *j* in iteration $\left(z+1\right)$ is updated as [9,26]:

(7) $$v_{j}^{\;\;z+1}=\omega_{z}\;v_{j}^{\;\;z}+c_{1}\;a_{2}\left(P_{best_{j}}-x_{j}^{\;\;z}\right)+c_{2}\;a_{3}\left(G_{best}-x_{j}^{\;\;z}\right)$$

where $\omega_{z}$ is the IW in the $z^{th}$ iteration, while $\;v_{j}^{\;z}\;\mathrm{and}\;x_{j}^{\;z}$ are the velocity and position of the particle *j* in the $z^{th}$ iteration, respectively. The best position of the particle *j* is denoted as $P_{best_{j}}$, while the global best position of all the particles is denoted as $G_{best}$. Additionally, $c_{1}$ and $c_{2}$ denote the acceleration parameters [9]. Moreover, $a_{2}$ and $a_{3}$ are two random numbers uniformly distributed between 0 and 1. It is noted that the particle $x_{j}$ is a binary vector and the velocity $v_{j}$ is also a vector. The sigmoid function $(Sig\left(v_{j}{\left(m\right)}^{z}\right)$) is given as [52,55]:

(8) $$Sig\left(v_{j}{\left(m\right)}^{z}\right)=\frac{1}{1+e^{-v_{j}{\left(m\right)}^{z}}}$$

Thus, the on/off state of the SC *m* in particle *j* in the *z*-th iteration is calculated as [52,55]:

(9) $$x_{j}{\left(m\right)}^{z}=\left\{\begin{array}{cc} 1, & a_{4}<Sig\left(v_{j}{\left(m\right)}^{z}\right)\\ 0, & otherwise \end{array}\right.$$

where $a_{4}$ is a random number uniformly distributed between 0 and 1.

Since the IW is the pivotal factor in the convergence of the PSO, it should be carefully adjusted. Thus, linearly increasing IW is utilized in this paper, where the IW linearly increases every iteration from $\omega_{min}$ to $\omega_{max\;}.\;$ The IW in the $z^{th}$ iteration is given as [33]:

(10) $$\omega_{z}=\left(\omega_{max\;}-\omega_{min}\right)\left(\frac{z-1}{Z_{max\;}-1}\right)+\omega_{min}\;$$

where $\omega_{max}$ and $\omega_{min}$ denote the maximum and minimum IW, respectively [33], and $Z_{max}$ is the maximum number of iterations [59].

## 4. Proposed Algorithms

To alleviate the number of active SCs and enhance the PE of the system, a linearly increasing IW-BPSO algorithm for SC on/off switching is proposed in this paper. Moreover, a novel SFR technique using CTs is proposed for SC sub-band allocation. The BPSO algorithm is applied first while the linearly increasing IW enhances the convergence of the algorithm. Then, the sub-bands are allocated to the active SCs using the novel SFR technique. It is worth noting that the new SFR technique is applied after the SC switching to allocate the sub-bands to the active SCs, only aiming to reduce the number of operations needed in the sub-band allocation to the SCs 

### 4.1. SC on/off Switching Using Linearly Increasing IW-BPSO Algorithm

In this paper, SC switching utilizing a linearly increasing IW-BPSO algorithm is proposed. The UE can associate with an SC *m* if $SINR_{k,m}$ exceeds a certain threshold ($SINR_{thr}$). At the beginning of our proposed algorithm, each UE *k* calculates the SINR from all SCs to determine all SCs that it can possibly associate with, then sorts the received SINR from these SCs in a descending order. The UE associates with the SC with the highest received SINR. If the number of UEs in this SC is larger than the maximum number of UEs in the SC ($U_{max}$), the UE connects with the SC having the next highest SINR. This continues until every UE is associated with one SC.

To minimize the number of active SCs and to elevate the PE, it is required to switch off the excessive SCs, taking into consideration the QoS of the UEs, since the SINR of every UE exceeds $SINR_{thr}$. Thus, a multi-objective optimization problem had to be solved; this problem can be written as:

(11) $$\mathrm{Objective}1:\;\min\;(\sum_{m=1}^{M}\gamma_{m})\;\mathrm{Objective}2:\;\max\;(PE_{sys})$$

subject to:

(11a) $$\gamma_{m},\;\in \;\{0,1\},\;\forall \;m\in M$$

(11b) $$\varphi_{m,k}\;\in \;\{0,1\},\;\forall \;m\in M,\;k\in U_{m}$$

(11c) $$\sum_{m=1}^{M}\varphi_{m,k}\;\gamma_{m}=1,\;\forall \;m\in M,\;k\in U_{m}$$

(11d) $$U_{m}\;\leq U_{max},\;\forall \;m\in M$$

where constraint (11a) is the on/off state indicator of the SC. Constraint (11b) is the UE association indicator. Constraint (11c) states that the UE is associated with only one active SC. Constraint (11d) indicates that the number of UEs in an SC cannot exceed the maximum number of UEs in an SC ($U_{max}$).

Algorithm 1 summarizes the proposed BPSO-based on/off SC switching algorithm. In line 1, after the random binary initialization of the population of every particle, the velocity of the particles is initialized as Equation (6). Then, for each iteration till the maximum number of iterations ($Z_{max}$) is reached, the IW ($\omega_{z}$) is computed (line 3) and the position ($x_{j}$) and velocity ($v_{j}$) of every particle *j* are updated using Equations (7) and (9), respectively (lines 5–6). Next, the fitness value of every particle *j* (F ($x_{j}$)) is computed using Equation (11) (line 7). If $F\;\left(x_{j}\right)<F\left(P_{best_{j}}\right),$ then $P_{best_{j}}=x_{j}$, and if $F\;\left(x_{j}\right)<F\left(G_{best}\right)\;$, then $G_{best}=x_{j}$ (lines 8–13).

**Algorithm 1:** Proposed linearly increasing IW-BPSO-based on/off SC switching.**Inputs:** Locations of UEs, locations of SCs, swarm size ($N_{par}$), maximum number of iterations $(Z_{max}$) **Output:** SC on/off indicator 1: **Initialize** the position ($x_{j}$) **randomly** and velocity ($v_{j}$) of every particle *j* as Equation (6). 2: **For** *z* = 1 to $Z_{max}$
3:  **Calculate**
$\omega_{z}$ using Equation (10) 4:  **For** each particle *j*
5:    **Update**
$v_{j}$ using Equation (7) 6:    **Update**
$x_{j}$ using Equation (9) 7:    **Calculate** new fitness value *F* ($x_{j}$) as Equation (5) 8:    **if** $F\;\left(x_{j}\right)<F\left(P_{best_{j}}\right)$
9:     $P_{best_{j}}\leftarrow \;x_{j}$ 10:    **end if**
11:    **if** $F\;\left(x_{j}\right)<F\left(G_{best}\right)$ 12:     $G_{best}\leftarrow x_{j}$ 13:    ** end if** 14:  **end For**
15: **end For**


### 4.2. SC Sub-Band Allotment Using Classification Trees (CTs)

**Figure 2:**
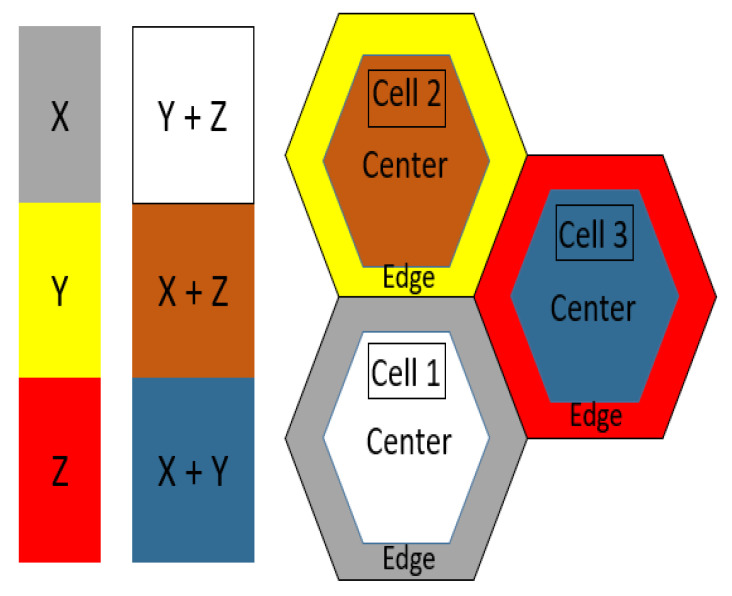
SFR example for hexagonal shaped cells with $N_{sub}$ = 3.

After the on/off switching decisions for all SCs are taken, the second phase is initialized, which is the sub-band allocation for the active SCs based on the SFR, which is illustrated in Figure 2, over three cells without loss of generality. In the shown example, if we have three hexagonal-shaped cells, each split into center and edge regions, the frequency band is divided to three ($N_{sub}$ = 3) sub-bands: X, Y, and Z. The edge region of cells 1, 2, and 3 are allocated sub-bands X, Y, and Z, respectively. Consequently, cell 1 is allocated sub-bands Y and Z in the center region. Similarly, cell 2 is allocated X and Z, and cell 3 is allocated X and Y. 

**Figure 3:**
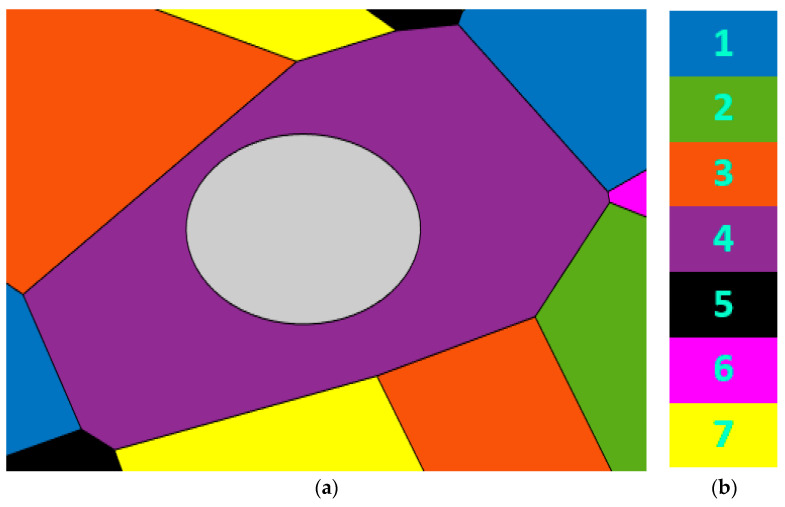
(**a**) A real example demonstrating a SC (the purple SC) with the center region (the grey region); (**b**) the seven used sub-bands.

In this technique, every SC is divided into center and edge regions. One of the $N_{sub}\;$ sub-bands can be used in the edge region of every SC, on condition that it is not used by the edge regions of the adjoin SCs. The center region of every SC can use the remaining sub-bands with reduced transmission power. This alleviates not only the interference to the adjoin SCs but also the power consumption of the whole network. To determine the vertices of the center region, the distance between the center of the SC and every vertex of the SC is computed. Then the distance between the center of the SC and the nearest SC vertex (the smallest distance) is determined and is regarded as the SC radius. The radius of the center zone is chosen as 50% of the SC radius as it maximizes the throughput of the system [13]. A real example is demonstrated in Figure 3a, displaying an SC (the purple SC) and its adjoining SCs. Figure 3b displays the seven sub-bands. Every SC uses one of the seven sub-bands in its edge region. While the center region of this SC (the grey region) can use the remaining six sub-bands. 

First, the SC senses the signals of the edge region of the adjoin SCs upon switching to obviate utilizing them. A binary indicator, $\tau_{m}=1,$ indicates the presence of unused sub-bands by the edge region of the adjoin SCs; otherwise, $\tau_{m}=0$. If there are unused sub-bands by the edge region of the adjoined SCs ($\tau_{m}=1$), then for every unused sub-band *f*, the distance between the SC and the closest nonadjacent SC using sub-band *f* in its edge region ($D_{Non-ad}{}_{f}$) is measured, and this is repeated for the rest of the remaining unused sub-bands. If all the sub-bands are used ($\tau_{m}=0$), then for every sub-band *q,* the distance between the SC and the adjoin SC using sub-band *q* in its edge region ($D_{Ad}{}_{q}$) is measured. Then, the edge region of the SC is allocated the sub-band used in the farthest adjoined SC. 

**Figure 4:**
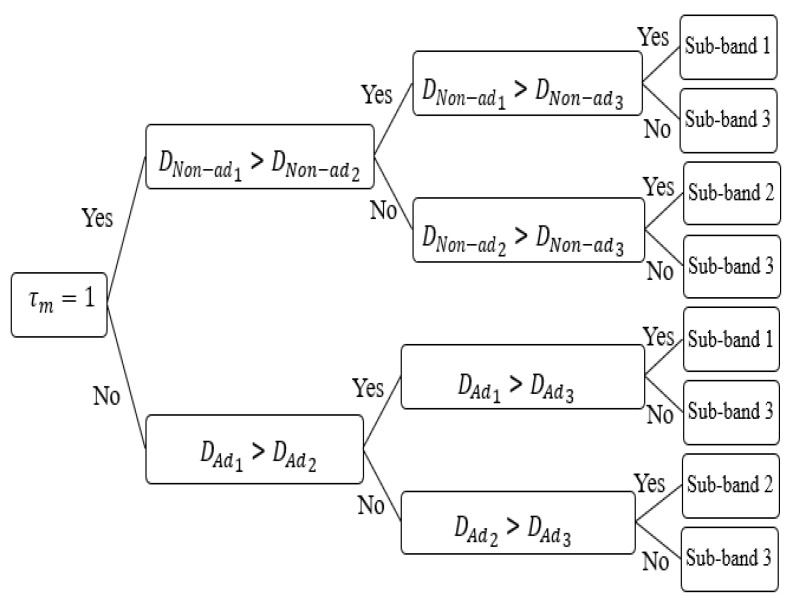
Classification tree (CT) example for three sub-bands.

According to $\tau_{m}$ and $D_{Non-ad}{}_{f}$ or $D_{Ad}{}_{q}$, the CT takes the decision to assign which sub-band to the edge region of the SC. A CT example of 3 sub-bands is shown in Figure 4. In case of the presence of unused sub-bands in the edge region of the adjoin SCs ($\tau_{m}=1$), then if the distance between the SC and the closest SC using the sub-band *1* in its edge region ($D_{Non-ad}{}_{f}=D_{Non-ad}{}_{1})\;$ is larger than $D_{Non-ad}{}_{2}$; $D_{Non-ad}{}_{1}$ and $D_{Non-ad}{}_{3}$ are checked. If $D_{Non-ad}{}_{1}$ is larger than $D_{Non-ad}{}_{3}$, then sub-band *1* is selected. In case all the sub-bands are utilized ($\tau_{m}=0$), then if the distance between the SC and the adjacent SC using sub-band *2* ($D_{Ad}{}_{q}=D_{Ad}{}_{2}$) is larger than $D_{Ad}{}_{1}$, then $D_{Ad}{}_{2}$ and $D_{Ad}{}_{3}$ are checked. If $D_{Ad}{}_{3}$ is larger than $D_{Ad}{}_{2}$, then sub-band *3* is selected. Afterward, the center region can utilize the remaining sub-bands with lower transmission power.

Unlike the computational complexity of the conventional SFR algorithm *O*(*S².* $N_{sub}$*²*), which depends on the network size (*S*) and the number of sub-bands ($N_{sub}$) [60], the computational complexity of the proposed CTs algorithm is much lower. Since the computational complexity of the DTs is *O(1)* [61], as no multiplication process is done and only a sequence of branching operations are performed, then computational complexity of the CTs is also *O(1)* for every SC. Thus, the computational complexity of the system is *S* x *O(1),* since only a sequence of branching operations are performed on moving along the CT according to the binary indicator *(*$\tau_{m})$ and the distance between the SC and the closest nonadjacent SC ($D_{Non-ad}{}_{f})$/farthest adjoining SC ($D_{{Ad}_{q}}$) using the sub-band in its edge region. Hence, applying SFR after the SC switching greatly reduces the computational complexity as SFR will be applied to the active SCs only (smaller *S*), while the complexity of the BPSO algorithm *O(*$Z_{max}\;$*. P)* depends on the maximum number of iterations and the population size (*P*) [62,63]. On the other hand, the average computational time of the proposed algorithm is less than one minute (54.857 s). In modern HetNets, the traffic load of the network is monitored for longer time periods in the order of 5–15 min [6,64,65,66], while the on-off switching decisions of the SCs are usually taken every 15–60 min [64,66]. Thus, the proposed algorithm is suitable for practical implementation in real time.

## 5. Numerical Results

The simulation parameters are presented in Table 1. Various Voronoi SCs are allocated in the coverage area of the MCs. The UEs are randomly deployed within the entire network. The simulations are performed utilizing MATLAB R2018. The swarm size is chosen in the range of 20–50 particles [9,67,68,69,70,71]. It is shown in [71] that 25 is the optimum size over 16 different sizes on 3 different cases. The IW is linearly increased from 0.4 to 0.9, which is the range recommended in [33,72,73]. The velocity of the particles is chosen in the range of [−0.6, 0.6] [9,74,75]. Adjusting these parameters is essential for the convergence of the BPSO algorithm. 

The performance of several algorithms is assessed, they are as follow:

- Always on: SFR is not utilized and all SCs are active.
- BPSO only: SFR is not utilized, but SC on/off switching is done via BPSO algorithm.
- Random 10%: SFR is not utilized, but 10% of the SCs are randomly chosen to be switched off. The remaining SCs are kept active.
- Proposed: the SC on/off switching is decided first using the BPSO algorithm, then the SFR is carried out using CTs.

**Figure 5:**
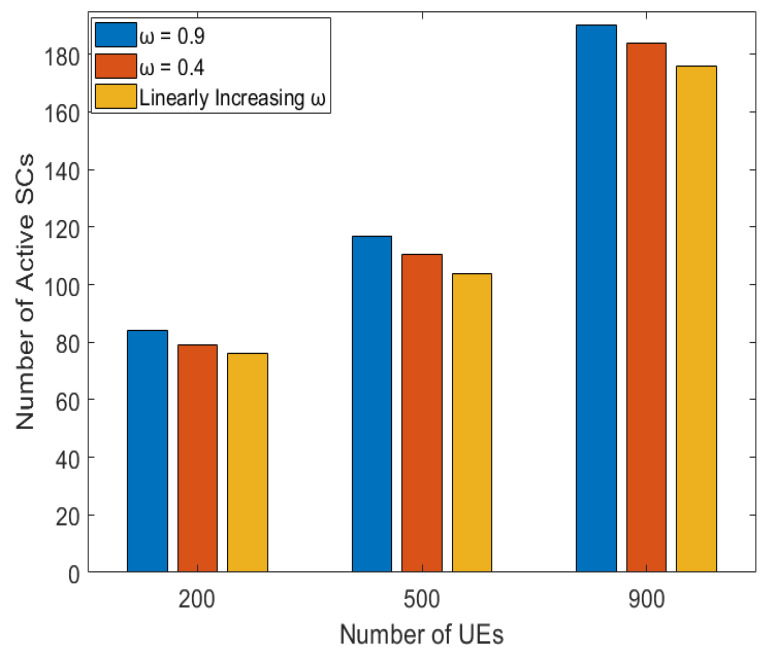
Number of active SCs for various numbers of UEs for different values of IW.

The number of active SCs for various number of UEs is demonstrated in Figure 5. The number of active SCs increases with increasing the number of UEs, as more SCs are being activated to guarantee the minimum required SINR of the UEs allowing the UEs to associate with the SC having the better SINR, enhancing their data rates and improving the system performance. The “ω = 0.4” has better convergence than the “ω = 0.9” for the 200, 500, and 900 UEs, respectively, since reducing the IW facilitates in exploring the search space (global search), while raising the IW aids in exploiting (local search) the search space. The “linearly increasing ω” shows better convergence than the fixed “ω = 0.9” and “ω = 0.4”, as in the “linearly increasing ω” case, the IW is lower at the beginning, allowing better exploration of the search space, and then it linearly increases, enhancing the local search. The fixed IW can get trapped during the search and is unable to find the global minimum number of active SCs. Therefore, the linearly increasing IW is chosen to be utilized in the proposed algorithm for the rest of the simulations in this paper.

**Figure 6:**
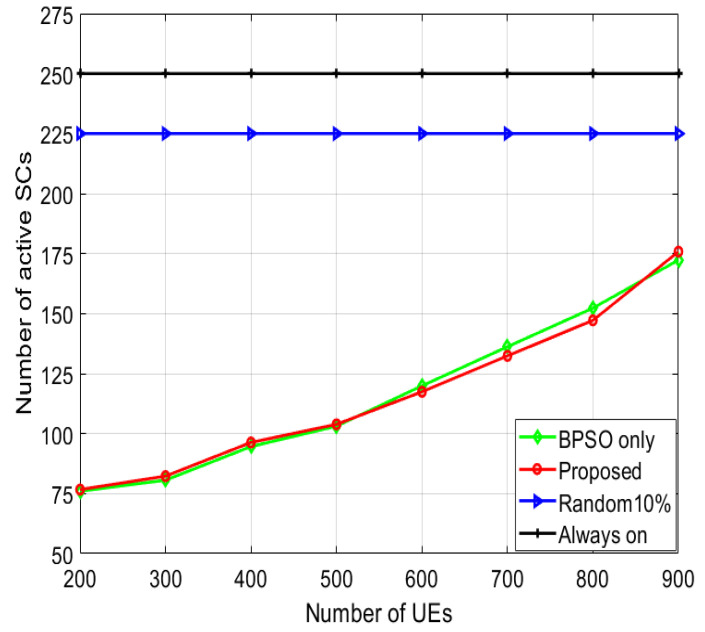
Number of active SCs for various numbers of UEs in case of linearly increasing IW.

The number of active SCs for various numbers of UEs is presented in Figure 6. The “always on” algorithm has the highest number of active SCs, since all the SCs are kept active. The “random 10%” algorithm has a lower number of active SCs than the “always on” algorithm, since in the “random 10%” algorithm, 10% of the SCs are randomly turned off. However, the number of active SCs is constant with regard to the number of UEs. The proposed and “BPSO only” algorithms have the least number of active SCs, as the SCs are turned off utilizing the BPSO algorithm. In both algorithms (BPSO only and proposed), BPSO is used for SC switching. That is why the number of active SCs of the two algorithms is very close. Moreover, the number of active SCs increases on increasing the number of UEs, since more SCs are switched on to sustain the minimum SINR of the UEs.

**Figure 7:**
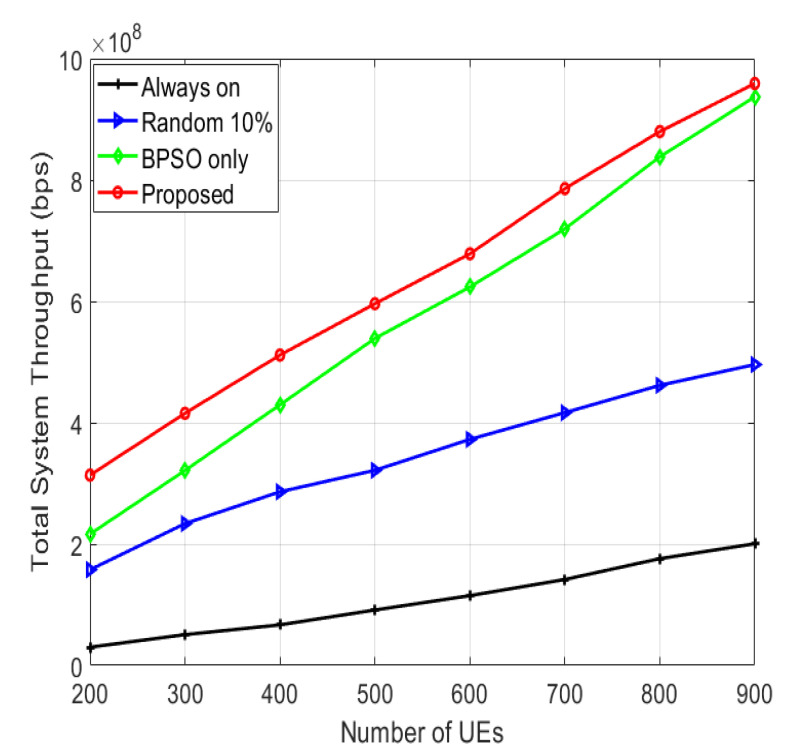
Total system throughput for various numbers of UEs.

The total system throughput for various numbers of UEs is shown in Figure 7. The proposed algorithm has the largest system throughput, since the proposed algorithm alleviates the interference levels, as it utilizes the SFR and switches the SCs on/off using the BPSO algorithm. The “BPSO only” algorithm has lower system throughput than the proposed algorithm because of its larger interference levels for not using SFR. The “random 10%” algorithm has lower system throughput than the “BPSO only” algorithm because of the random selection of the switched-off SCs, which is not the optimum one. The “always on” algorithm has the minimum system throughput because it has the largest interference levels as all the SCs are continuously active and it does not utilize the SFR.

**Figure 8:**
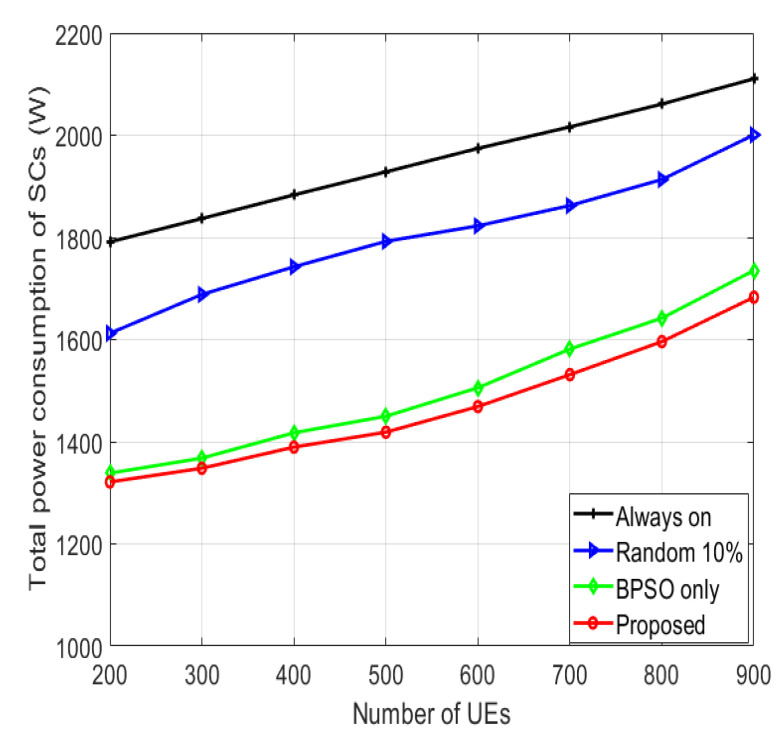
Total system power consumption for various numbers of UEs.

The total power consumption of the system for various numbers of UEs is presented in Figure 8. The “always on” algorithm has the largest power consumption since no switching-off techniques are used. The “random 10%” algorithm has less power consumption than the “always on” algorithm because of the power savings from the switched-off SCs and higher power consumption than the “BPSO only” algorithm. The proposed algorithm consumes the least power because of the turning-off of the SCs utilizing BPSO, then applying the SFR minimizing the power consumption furthermore, because of the reduced transmission power for the UEs in the center region. In the proposed algorithm, the power consumption increases with the increment of the number of UEs, as more SCs are kept active to ensure the QoS of the UEs.

**Figure 9:**
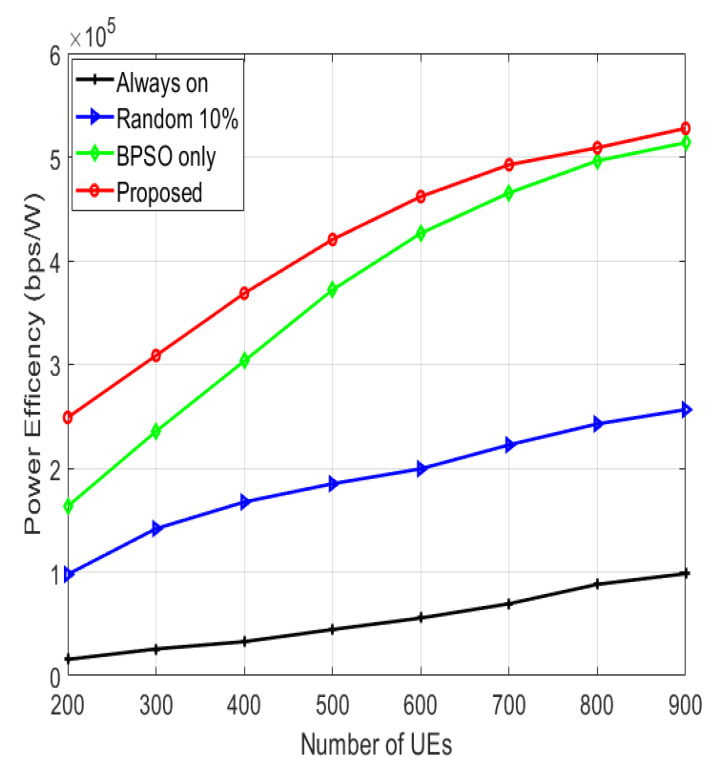
Power efficiency for various numbers of UEs.

The PE of the system for various numbers of UEs is demonstrated in Figure 9. The proposed algorithm has the highest PE because of the reduced power consumption and the elevated system throughput. The “BPSO only” algorithm has lower PE because of its alleviated throughput and higher system power consumption compared to the proposed algorithm. The “random 10%” has lower PE than the “BPSO only” algorithm because of its alleviated system throughput caused by the random choice of the switched-off SCs. The “always on” algorithm has the least PE, since it has the largest power consumption and the least data rates because all the SCs are continuously active.

**Figure 10:**
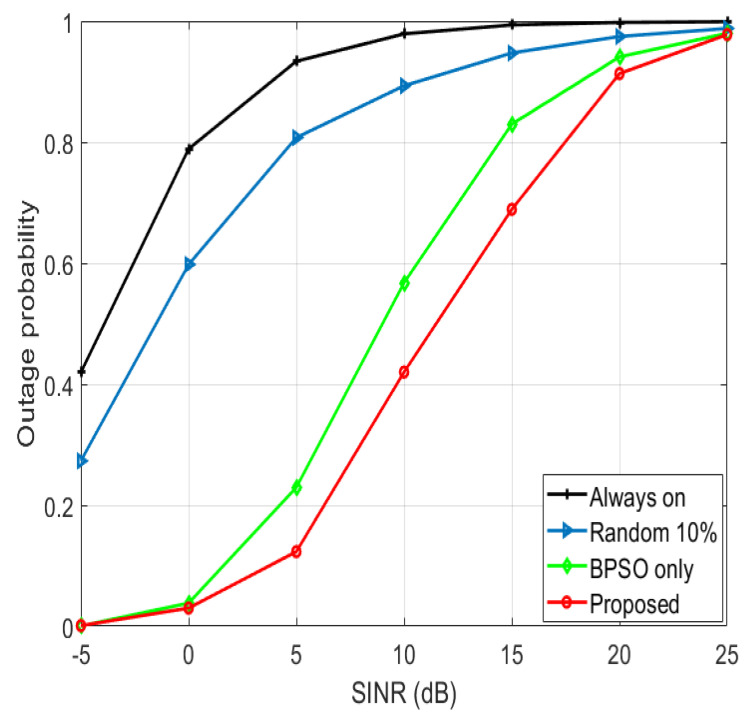
Outage probability for various SINR thresholds in the case of 900 UEs.

The outage probability for various SINR thresholds in the case of 900 UEs is depicted in Figure 10. The outage probability is defined as the percentage of UEs that are unable to attain a certain SINR threshold. The proposed algorithm has the minimum outage probability because of the enhanced SINR of the users, which resulted from minimizing the interference. The “BPSO only” algorithm has larger outage probability than the proposed algorithm, since it has larger interference levels. Both algorithms consider the QoS of the users, as all the users have SINR larger than $SINR_{thr}$ in both algorithms. The “random 10%” algorithm has larger outage probability than the “BPSO only” because of the deteriorated system performance. The “always on” algorithm has the largest outage probability because of the huge interference levels, as the SFR principle is not utilized and no on/off switching techniques are used, diminishing the total performance of the system.

## 6. Conclusions

Minimizing the power consumption of the SCs and elevating the PE of the network are huge challenges facing the 5G HetNets. In this paper, to tackle these challenges, novel algorithms are proposed based on linear increasing IW-BPSO and SFR. The BPSO algorithm is used for SC on/off switching reducing the power consumption of the system without deteriorating the QoS of the UEs. Moreover, the linearly increasing IW is exploited to enhance the convergence of the BPSO algorithm to find the minimum number of active SCs. Furthermore, the CT-based SFR is proposed, where the SCs are divided into center and edge regions and different sub-bands are allocated to the edge regions of the adjoining SCs, minimizing the interference among the SCs. The results demonstrate that the proposed algorithms surpass the other conventional algorithms with regard to the total system power consumption, the total system throughput, the PE, and the outage probability. Additional work can be done in the future to address the coverage hole problem in Voronoi cells and to enhance the accuracy of the PSO algorithm using dynamic inertia weight.

## Figures and Tables

**Table 1:** Simulation Parameters [9,13,14,33,59].

Parameters	Value
SC transmission power [13]	SFR: 20 dBm(center), 22 dBm(edge) No SFR: 22 dBm
SC baseline power ($P_{m_{on}}$) [14]	6.8 W
Maximum number of UEs in the SC ($U_{max}$) [9]	30
$\mathrm{Swarm}\;\mathrm{size}\;(N_{par}$) [9]	25
Maximum IW ($\omega_{max}$) [33]	0.9
Minimum IW ($\omega_{min}$) [33]	0.4
Maximum velocity of the particle ($v_{max}$) [9]	0.6
Minimum velocity of the particle ($v_{min}$) [9]	−0.6
Maximum number of iterations ($Z_{max}$) [59]	500
Total bandwidth [13]	20 MHz
RB bandwidth [13]	180 KHz
Maximum number of RBs [13]	106
Number of sub-bands ($N_{sub}$) [13]	7
Noise power spectral density [14]	−174 dBm/Hz
SINR threshold ($SINR_{thr}$) [9]	−5 dB
SC inactive level (β) [14]	0.63
Portion of power consumption due to the feeder losses and power amplifier (θ) [14]	4

## Nomenclature

**Symbol**	**Description**
$a_{1},a_{2},\;a_{3}$ and $a_{4}$	Random numbers uniformly distributed between 0 and 1
$B_{k}$	Bandwidth allocated to UE *k*
$B_{RB}$	Resource block (RB) bandwidth
$c_{1}$ and $c_{2}$	Acceleration parameters
$C_{sys}$	Total throughput of the system
$D_{f}$	Distance between the SC and the closest SC using sub-band *f* in its edge region
$d_{m,k}$	Distance between SC *m* and UE *k*
$G_{m,k}$	Channel gain between UE *k* and SC *m*
$G_{best}$	Global best position of all the particles
$L_{k,m}$	Number of requisite RBs for UE *k* in SC *m* to achieve the minimum data rate
*M*	Number of SCs
N	Noise power
$P_{best_{j}}$	Best position of the particle *j*
$P_{m}$	Transmission power of SC *m*
$P_{m_{tot}}$	Total power consumption of SC *m*
$P_{m_{tx}}$	Transmission power consumption of SC *m*
$P_{sys}$	Total power consumption of the system
$PE_{sys}$	PE (power efficency) of the system
$R_{k,m}$	Data rate of UE *k* in SC *m*
$SINR_{k,m}$	Signal to interference noise ratio of UE *k* in SC *m*
$U_{m}$	Number of UEs in SC *m*
$v_{j}^{\;\;z}$	Velocity of the particle *j* in the $z^{th}$ iteration
$\omega_{z}$	Inertia Weight (IW) in the $z^{th}$ iteration
$x_{j}^{\;\;z}$	Position of the particle *j* in the $z^{th}$ iteration
*α*	Path loss exponent
$\gamma_{m}$	SC *m* on/off indicator
$\varphi_{m,k}$	UE *k* association indicator with SC *m*
